# Magnetic Multi-Enzymatic System for Cladribine Manufacturing

**DOI:** 10.3390/ijms232113634

**Published:** 2022-11-07

**Authors:** Guillermo Cruz, Laura Pilar Saiz, Muhammad Bilal, Lobna Eltoukhy, Christoph Loderer, Jesús Fernández-Lucas

**Affiliations:** 1 Applied Biotechnology Group, Universidad Europea de Madrid, Urbanización El Bosque, Calle Tajo, s/n, 28670 Villaviciosa de Odón, Spain; 2 Institute of Chemical Technology and Engineering, Faculty of Chemical Technology, Poznan University of Technology, Berdychowo 4, PL-60695 Poznan, Poland; 3 Chair of Molecular Biotechnology, Institute for Microbiology, Technische Universität Dresden, Zellescher Weg 20b, 01217 Dresden, Germany

**Keywords:** cascade synthesis, magnetic catalysts, enzyme immobilization, transglycosylation reaction, nucleoside analogues

## Abstract

Enzyme-mediated processes have proven to be a valuable and sustainable alternative to traditional chemical methods. In this regard, the use of multi-enzymatic systems enables the realization of complex synthetic schemes, while also introducing a number of additional advantages, including the conversion of reversible reactions into irreversible processes, the partial or complete elimination of product inhibition problems, and the minimization of undesirable by-products. In addition, the immobilization of biocatalysts on magnetic supports allows for easy reusability and streamlines the downstream process. Herein we have developed a cascade system for cladribine synthesis based on the sequential action of two magnetic biocatalysts. For that purpose, purine 2′-deoxyribosyltransferase from *Leishmania mexicana* (*Lm*PDT) and *Escherichia coli* hypoxanthine phosphoribosyltransferase (*Ec*HPRT) were immobilized onto Ni^2+^-prechelated magnetic microspheres (MagReSyn^®^NTA). Among the resulting derivatives, M*Lm*PDT3 (activity: 11,935 IU/g_support_, 63% retained activity, operational conditions: 40 °C and pH 5–7) and M*Ec*HPRT3 (12,840 IU/g_support_, 45% retained activity, operational conditions: pH 5–8 and 40–60 °C) emerge as optimal catalysts for further synthetic application. Moreover, the M*Lm*PDT3/M*Ec*HPRT3 system was biochemically characterized and successfully applied to the one-pot synthesis of cladribine under various conditions. This methodology not only displayed a 1.67-fold improvement in cladribine synthesis (compared to M*Lm*PDT3), but it also implied a practically complete transformation of the undesired by-product into a high-added-value product (90% conversion of Hyp into IMP). Finally, M*Lm*PDT3/M*Ec*HPRT3 was reused for 16 cycles, which displayed a 75% retained activity.

## 1. Introduction

Nowadays, the partial or total replacement of environmentally unfriendly and tedious chemical procedures by enzyme-mediated processes is considered common practice in pharma industries [1,2,3]. Enzymatic synthesis of nucleic acid derivatives (nucleosides and nucleotides) is a clear example of how biocatalysis offers an eco-friendly, regio-, stereo-, and enantioselective alternative to traditional multi-step and environmentally harmful chemical processes [4,5,6,7]. To this effect, a wide variety of enzymes have been employed as practical catalysts for the mono and multi-enzymatic synthesis of NAs, including nucleoside phosphorylases [8,9], 2′-deoxyribosyltransferases [4,5,10], phosphoribosyltransferases [11,12,13], and nucleoside kinases [14,15,16], among others.

Cladribine (Mavenclad, 2-chloro-2′-deoxyadenosine) is a clinical drug for the treatment of hairy cell lymphoid and myeloid leukemia [17] which has also received EMA and FDA approval for the treatment of relapsing–remitting multiple sclerosis (RRMS) [18]. Due to its broad clinical applicability, the development of novel, efficient and eco-friendly alternatives to classical chemical methods are gaining ground. Among them, bioprocesses based on enzymatic transglycosylation from a nucleoside donor to a nucleobase acceptor catalyzed by 2′-deoxyribosyltransferases (NDTs), or nucleoside phosphorylases (NPs), are the most habitual strategies [19,20,21,22,23,24,25,26]. Of these, we focused our attention on NDTs. 

Traditionally, NDTs were classified as type I NDTs (PDT), which perform the transglycosylation reaction between purines; and type II NDTs (NDT), which act on pyrimidines and purines [4,5]. Interestingly, NDTs display high chemo-, regio-, and/or enantioselectivity while converting a broad range of natural and modified bases [4,5]. However, despite this enzymatic approach vastly surpassing chemical procedures, in terms of efficiency (one-pot regio- and enantioselective reaction), or sustainability (green conditions, aqueous buffers), several operational constraints must be addressed from an industrial perspective. 

A major problem that hinders the industrial implementation of the bioprocesses is the high costs involved in the production and purification of recombinant enzymes. In this respect, enzyme immobilization offers the possibility to reuse the biocatalysts and, therefore, overcome this undesired operational constraint. A comprehensive design of the immobilization process could modify or enhance various enzyme properties, such as thermal stability, activity, and specificity [27,28,29,30]. The use of magnetic matrices (micro or macro size) as supports for enzyme immobilization provides other additional advantages, such as an easy biocatalyst recovery and a simplification in the downstream process. Moreover, the use of magnetic stirring avoids any deterioration of the biocatalysts caused by mechanical agitation [31,32]. Each of these simplifies the operational processing and increases efficiency, leading to more cost-effective processes.

**Figure 1:**
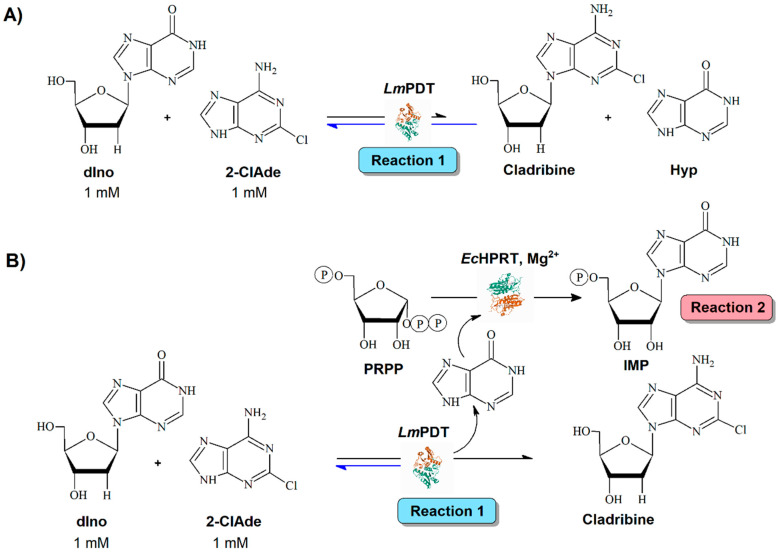
Enzymatic synthesis of Cladribine catalyzed by: (**A**) *Lm*PDT. (**B**) *Lm*PDT/*Ec*HPRT. dIno: 2′-deoxyinosine; 2-ClAde: 2-chloroadenine; Hyp: hypoxanthine; PRPP: 5-phospho-α-D-ribosyl-1-pyrophosphate; *Lm*PDT: *Leishmania mexicana* purine 2′-deoxyribosyltransferase; *Ec*HPRT; *Escherichia coli* hypoxanthine phosphoribosyltransferase.

Equally important is the minimization of undesired by-products (those without economic interest) to simplify the downstream procedure. As a result of the transglycosylation (reaction 1), a second nucleobase is released into the reaction mixture, complicating the purification of the main product (Figure 1A). Moreover, if we consider that, most of the time, the reaction equilibrium in transglycosylation is not shifted towards product formation, up to four molecules are present in the solution. This involves an additional drawback for the purification and further processing. Le Chatelier’s principle states that a change in pressure, temperature, or concentration of products or reactants makes the equilibrium shift in the opposite direction to offset the change. As a consequence, a modification of the initial nucleoside/base ratio, or the conversion of the released nucleobase into other added-value molecules (inosine-5′-monophosphate, IMP) by a second enzyme (reaction 2), may contribute to a shift in the equilibrium of the main reaction (Figure 1B) [24].

In previous work, the sequential action of NDT from *Lactobacillus delbrueckii* and *Thermus thermophilus* HGXPRT demonstrated the feasibility of this cascade system [24]. Unfortunately, several operational problems, such as the partial conversion of nucleobase released into NMP by the *Tt*HGPXRT, a shift of the equilibrium of the transglycosylation reaction below expectations, the mandatory production and purification of required enzymes, and the difficulty in separating the catalysts from the reaction medium, forced us to redesign and improve this cascade system.

Since the biocatalyst reusability and the absence of by-products are essential requisites for the industrial scale-up, herein we report the development of a magnetic multi-enzymatic system for cladribine manufacturing based on the sequential action of *Leishmania mexicana* purine 2′-deoxyribosyltransferase (*Lm*PDT) [33] and *E. coli* K12 hypoxanthine phosphoribosyltransferase (*Ec*HPRT) [34,35] (Figure 1).

To this end, *Lm*PDT and *Ec*HPRT were immobilized onto Ni^2+^-prechelated magnetic microspheres. The resulting magnetic catalysts (M*Lm*PDT and M*Ec*HPRT derivatives) were further optimized (catalyst loading, activity recovery) and biochemically characterized (effect of pH and T on enzyme activity). Among the different derivatives, M*Lm*PDT3 (activity: 11,935 IU/g_support_, 63% retained activity, operational conditions: 40 °C and pH 5–7) and M*Ec*HPRT3 (12,840 IU/g_support_, 45% retained activity, operational conditions: pH 5–8 and 40–60 °C) were selected as candidates for the envisioned magnetic multi-enzymatic system, M*Lm*PDT/M*Ec*HPRT. Finally, the proposed magnetic multi-enzymatic system, M*Lm*PDT3/M*Ec*HPRT3, was assayed on the one-pot synthesis of cladribine from 2′-deoxyinosine (dIno) and 2-chloroadenine (2-ClAde) (Figure 1). As a major advantage, M*Lm*PDT3/M*Ec*HPRT3 displayed a 1.67-fold improvement of conversion in cladribine synthesis (referring to M*Lm*PDT3), and it also allowed for the nearly total transformation (>90%) of the released nucleobase (an undesired by-product) into a second, high-added-value product (inosine-5′-monophosphate). Moreover, the effect of different parameters (pH, temperature, time course, M*Lm*PDT3/M*Ec*HPRT3 ratio) on cladribine production was also studied, displaying 40 °C, pH 7.0, 1.2 µg/0.4 µg (M*Lm*PDT3/M*Ec*HPRT3 ratio) and 10 min as optimal conditions. Finally, the magnetic multi-enzymatic system was reused for up to 16 cycles, maintaining ≈75% of initial activity.

## 2. Results and Discussion

### 2.1. Enzyme Immobilization

As previously reported, the immobilization of His-tagged proteins onto Ni^2+^ pre-chelated matrices usually leads to a non-aggressive and oriented immobilization process which does not usually affect the enzyme activity [36,37]. On this basis, His-tagged *Lm*PDT and His-tagged *Ec*HPRT were immobilized onto Ni^2+^-chelated Fe_2_O_3_ porous microspheres (MagReSyn^®^NTA).

As shown in Table S1 (supplementary data), the preliminary catalyst load experiments (enzyme/support mass ratio) displayed M*Lm*PDT3 (activity: 11,935 IU g^−1^_biocat_ dAdo synthesis; activity recovery of 63%) as the strongest candidate for further experiments. Interestingly, M*Lm*PDT3 displayed activity superior to other reported immobilized NDT derivatives, such as mutant PDT from *Trypanosoma brucei* NDT (*Tb*PDT) immobilized onto Ni^2+^ pre-chelated spheres (M*Tb*PDT_V11S_; 10,552 IU g^−1^; retained activity 54%) [37], wild type *Tb*PDT immobilized onto glutaraldehyde-activated Fe_2_O_3_ microspheres (M*Tb*PDT; 4200 IU g^−1^; retained activity 22%) [25], wild-type *Lactobacillus reuteri* NDT covalently immobilized onto epoxy-activated beads EC-EP303 (S*Lr*NDT4; 65.4 IU g^−1^; retained activity 13.5%) [38,39], and onto glutaraldehyde activated magnetic chitosan beads (15 IU g^−1^; retained activity 27%) [40], or wild type *Bacillus pshychrosaccharolyticus* NDT immobilized onto PEI-functionalized agarose, cross-linked with aldehyde-dextran (2.2 IU g^−1^; retained activity 36%) [41,42]. 

Furthermore, among the M*Ec*HPRT derivatives, M*Ec*HPRT3 was chosen as the optimal derivative (Table S1) (activity: 12,840 IU g^−1^_biocat_ in IMP synthesis; activity recovery of 45%). As happened with M*Lm*PDT3, M*Ec*HPRT3 displayed activity superior to other previously immobilized purine PRTs, such as HGXPRT or APRT from *Thermus thermophilus* covalently immobilized onto glutaraldehyde-activated Fe_2_O_3_ microspheres (M*Tt*HGXPRT3, activity 1581, IU g^−1^_biocat_, retained activity 29%; M*Tt*APRT2B, activity 480 IU g^−1^_biocat_, retained activity 52%; M*Tt*APRT2B, activity 507 IU g^−1^_biocat_, retained activity 44%) [13,43]. 

### 2.2. Biochemical Characterization of Immobilized Derivatives

**Figure 2:**
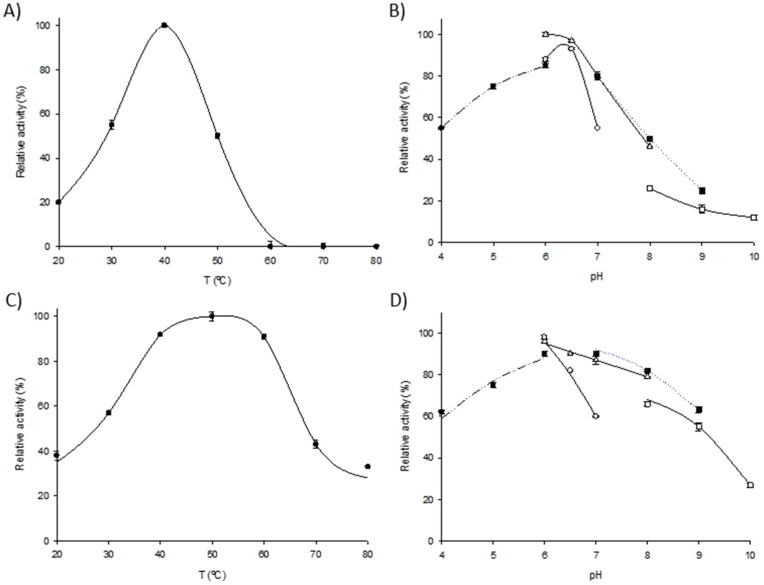
Biochemical characterization of M*Lm*PDT3 and M*Ec*HPRT3. (**A**) Effect of temperature on M*Lm*PDT3 activity (**●**). (**B**) Effect of pH on M*Lm*PDT3 activity, (**●**) 50 mM sodium citrate (pH 4–6), (△) 50 mM sodium phosphate (pH 6–8), (○) 50 mM MES (pH 6–7), (■) 50 mM Tris-HCl (pH 7–9), (□) 50 mM sodium borate (pH 8–10). (**C**) Effect of temperature M*Ec*HPRT3 activity (**●**). (**D**) Effect of pH on M*Ec*HPRT3 activity, (**●**) 12 mM sodium citrate (pH 4–6), (△) 12 mM sodium phosphate (pH 6–8), (○) 12 mM MES (pH 6–7), (■) 12 mM Tris-HCl (pH 7–9), (□) 12 mM sodium borate (pH 8–10).

To determine the most appropriate operational condition for implementing the magnetic M*Lm*PDT/M*Ec*HPRT system, the effect of pH and temperature on catalyst activity was assayed for both derivatives separately. M*Lm*PDT3 displayed maximum activity when incubated in 50 mM MES buffer, pH 6.0 at 40 °C (Figure 2A,B). However, it must be highlighted that M*Lm*PDT3 also displayed higher activity (>75%) in the pH range 6–7. Regarding M*Ec*HPRT3, the magnetic derivative displayed maximum activity at 50 °C. M*Ec*HPRT3 also showed high activity (>80%) in a broad range of temperatures (40–60 °C) and pH (5–7). Based on these preliminary results, pH 6.0–7.0 and 40 °C were initially selected as starting conditions for the further implementation of M*Lm*PDT3/M*Ec*HPRT3. 

### 2.3. Biochemical Characterization of the MLmPDT3/MEcHPRT3 System

**Figure 3:**
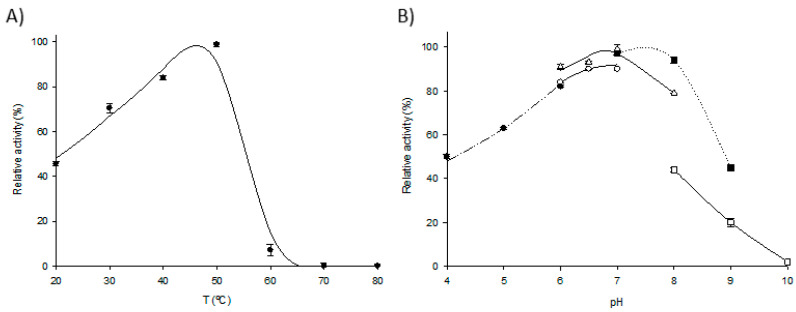
Biochemical characterization of the M*Lm*PDT3/M*Ec*HPRT3 system. (**A**) Effect of temperature on cladribine synthesis (●). (**B**) Effect of pH on cladribine synthesis, (**●**) 50 mM sodium citrate (pH 4–6), (△) 50 mM sodium phosphate (pH 6–8), (○) 50 mM MES (pH 6–7), (■) 50 mM Tris-HCl (pH 7–9), (□) 50 mM sodium borate (pH 8–10).

Once the preliminary operational conditions were proposed, we assayed the effects of pH and temperature on the M*Lm*PDT3/M*Ec*HPRT3 system to determine the optimal conditions for cladribine synthesis. As shown in Figure 3A, the multi-enzymatic systems exhibit >80% relative activity in a temperature range of 40 to 50 °C, with a peak at 50 °C. Although M*Lm*PDT3 suffered a significant activity loss at temperature values above 40 °C (Figure 2A), M*Ec*HPRT3 was found to be active (>90% relative activity) in the 40–60 °C range, with a peak at 50 °C (Figure 2C). Overall, the M*Lm*PDT3/M*Ec*HPRT3 system possesses a higher operability (compared to M*Lm*PDT3). This is probably associated with the conversion of the released Hyp into IMP, which contributes to shifting the equilibrium of the main reaction towards cladribine production.

Regarding the effect of pH on the activity of the cascade system, the experimental results are in line with the consensus conditions previously predicted (see above), displaying optimal activity when 50 mM Tris-HCl or 50 mM sodium phosphate were used as reaction buffers. However, due to the phosphate buffer’s capacity to react with some divalent metal cations (leading to enzyme inhibition), including Ca^2+^ and Mg^2+^, we selected Tris-HCl pH 7 as the optimal reaction buffer for further thermostability studies.

### 2.4. Thermal Stability of the Magnetic MLmPDT3/MEcHPRT3 System 

One of the most essential features to be considered in the deployment of enzymes for large-scale industrial applications is the stability of biocatalysts under operational conditions. In this way, selecting non-appropriate reaction conditions may lead to lower enzyme stability, a shorter lifespan, and poor catalytic performance. To this end, we assay the operational stability of the immobilized M*Lm*PDT3/M*Ec*HPRT3 system when stored at 40 °C and 50 °C in 50 mM Tris-HCl, pH 7. 

**Figure 4:**
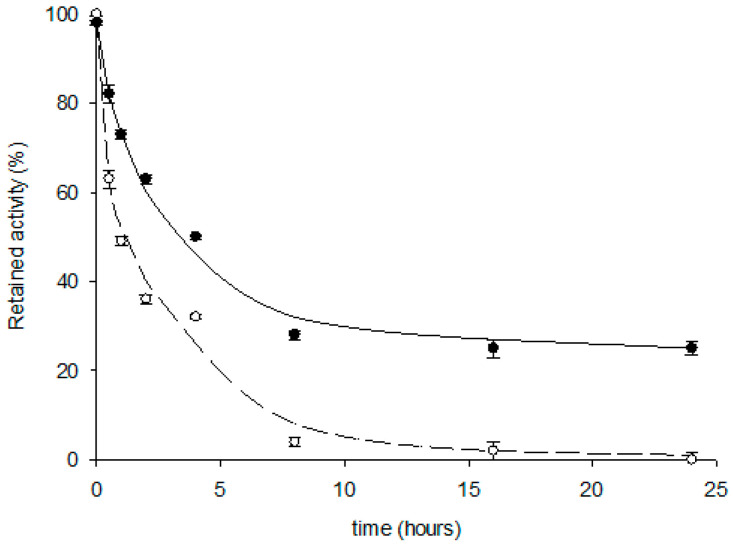
Thermal inactivation profile of M*Lm*PDT3/M*Ec*HPRT3 on cladribine synthesis at 40 °C (**●**) and 50 °C (○).

As shown in Figure 4, a four-fold enhanced, long-term stability is observed when stored at 40 °C (t_1/2_^40 °C^ ≈ 4 h, t_1/2_^50 °C^ ≈ 1 h). Thus, even though previous preliminary results suggested selecting 50 mM Tris-HCl, pH 7, and 50 °C (Figure 3) as the most appropriate reaction conditions, the thermal study of magnetic M*Lm*PDT3/M*Ec*HPRT3 revealed 50 mM Tris-HCl pH 7 and 40 °C as optimal conditions (Figure 4).

Although enzyme immobilization has been extensively used to improve the stability of biocatalysts, the developed M*Lm*PDT3/M*Ec*HPRT3 system displayed relatively low thermostability. In fact, as previously reported, soluble *Lm*PDT displayed higher thermostability (100% retained activity when stored in 50 mM MES buffer, pH 6.5 at 40 °C) when compared to the multi-enzymatic system. Given these results, the immobilization process through IMAC methodologies could affect the multimeric assembly of the enzyme, triggering the disassociation of the subunits [29,44,45]. In this sense, subunit disassociation stands out as a complex issue causing enzyme inactivation and therefore affects enzyme stability. Moreover, since matrices for IMAC immobilization are not physically inert, different multi-punctual adsorptions (hydrophobicity or ionic interactions, among others) between the protein and support can also occur, affecting protein folding and, thus, the biocatalyst stability [29,44,45].

### 2.5. Application of the MLmPDT3/MEcHPRT3 System in Manufacturing Cladribine and IMP

Despite the enzymatic synthesis of Cladribine catalyzed by NDTs or NPs having been extensively described in publications [19,20,21,22,23,24,25,26], the presence of released nucleobase (reaction 1, Figure 1) is an operational constraint that hampers the final downstream processing, and therefore, the industrial implementation of the process. Herein we propose a tentative solution for this operational drawback based on applying a magnetic cascade system. To this end, we initially compared the transglycosylation capability of M*Lm*PDT3/M*Ec*HPRT3 and M*Lm*PDT3 on cladribine synthesis from dIno (2′-deoxyribose donor) and 2-ClAde (acceptor) (Table 1) (reaction 1); and additionally, we quantified the IMP formation (reaction 2) from released Hyp (acceptor) and PRPP (ribose-5′-phosphate donor). The experimental results revealed a 1.3-fold enhancement in cladribine production (Table 1) and, more interestingly, a 75% conversion of released Hyp into IMP, an added-value compound used as an additive in the food industry [12,46].

**Figure 5:**
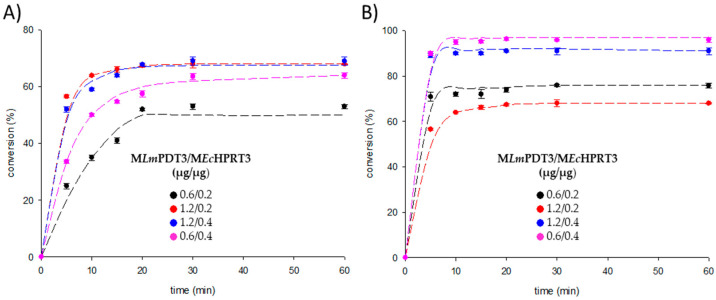
Effect of M*Lm*PDT3/M*Ec*HPRT3 ratio (μg of immobilized *Lm*PDT/μg of immobilized *Ec*HPRT) in the product formation. (**A**) cladribine (reaction 1). (**B**) Hyp converted into IMP (reaction 2).

After verifying the consistency of the initial hypothesis, the next step was to evaluate the influence of different operational parameters affecting the potential scale-up of the bioprocess. To start with, different time-course curves at different M*Lm*PDT3/M*Ec*HPRT3 ratios were performed under the previously determined reaction conditions (Figure 5).

As shown in Figure 5, ratios 1.2/0.4 and 1.2/0.2 (M*Lm*PDT3/M*Ec*HPRT3) lead to a 1.67-fold increase of cladribine production at 20 min (referred to M*Lm*PDT3, Table 1). Additionally, a 90% conversion of released Hyp into IMP was observed when using a 1.2/0.4 ratio, which considerably improved (up 1.2-fold) results displayed for the initial conditions (0.6/0.2 ratio, 75%) (Table 1). Interestingly, the experimental results revealed no significant differences at 10 min (59% conversion of dIno into cladribine, 90% conversion of released Hyp into IMP). Considering these features, the 1.2/0.4 ratio and 10 min were selected as the optimal conditions for the reusability experiments.

**Figure 6:**
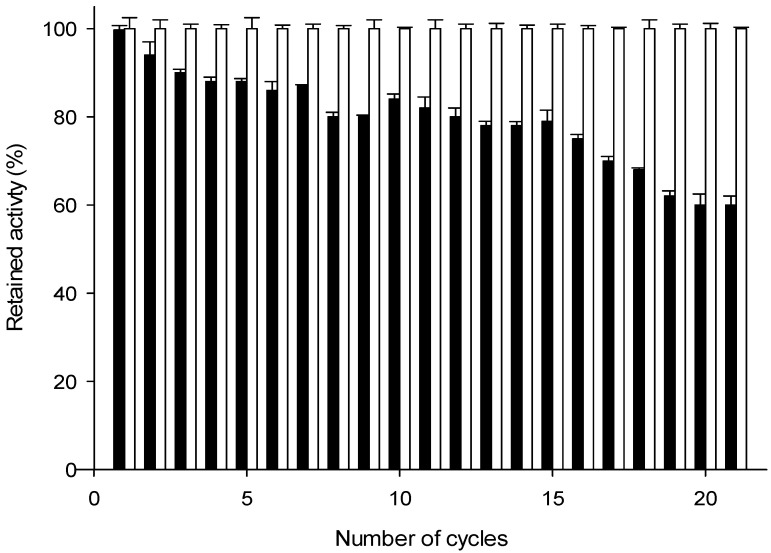
Reusability of the M*Lm*PDT3/M*Ec*HPRT3 system. 2′-deoxyribosyltransferase activity, reaction 1 (black bar); phosphoribosyltransferase activity, reaction 2 (white bar).

As some of the major drawbacks for the industrial implementation of bioprocesses are the high cost of producing enzymes and the difficulty in recovering and reusing the biocatalyst, the reusability of the magnetic M*Lm*PDT3/M*Ec*HPRT3 system was addressed (Figure 5). As shown in Figure 6, the magnetic M*Lm*PDT3/M*Ec*HPRT3 system maintained around 75% of the initial 2′-deoxyribosyltransferase activity (reaction 1, cladribine synthesis) during 16 cycles and also displayed around 60% of retained activity after 21 cycles. These results conform with experimental results derived from thermal inactivation curves (t_1/2_^40 °C^ ≈ 4 h, Figure 4).

It must be noted that no loss of phosphoribosyltransferase activity (reaction 2, IMP production) was observed, suggesting M*Ec*HPRT3 remains stable under operational conditions. So, according to experimental data, an improvement of M*Lm*PDT3 stability may lead to an increase in the long-term stability of the cascade system (ongoing work). 

## 3. Materials and Methods

### 3.1. Materials

Cell culture medium reagents were from Difco (St. Louis, United States). Moreover, HPLC solvents and buffers were purchased from Sigma-Aldrich (Madrid, Spain). All other reagents, nucleosides, and nucleobases used in this work were provided by Carbosynth Ltd. (Compton, UK).

### 3.2. Gene Expression and Protein Purification

All the DNA constructions were obtained from Genscript (Piscataway, United States) as *Nde*I-*Eco*RI fragments subcloned into the expression vector pET28b(+). The recombinant plasmids were used to transform *E. coli* BL21 (DE3) cells under the conditions described elsewhere [47]. Moreover, the overexpression of *Leishmania mexicana* His-tag NDT (UniProtKB E9AWJ0) and *Escherichia coli* His-tag HPRT (UniProtKB P0A9M2), and the cell extract preparation for protein purification were performed following the previous protocol reported by Acosta et al. [24,47]. 

The cleared lysates were loaded onto a 5-mL HisTrap FF column (GE Healthcare), pre-equilibrated in a binding buffer (20 mM Tris-HCl buffer, pH 7.5, with 100 mM NaCl and 20 mM imidazole) using an ÄKTA Prime FPLC (GE Healthcare). Bound proteins were eluted using a linear gradient of imidazole (20–500 mM). Fractions containing proteins of interest were identified by SDS-PAGE, pooled, concentrated, and loaded onto a HiLoad 16/60 Superdex 200 prep grade column (GE Healthcare) pre-equilibrated in 20 mM sodium phosphate, pH 7. Fractions with the protein of interest were identified by SDS-PAGE (Figure S1). The protein concentration was determined spectrophotometrically by UV absorption 280 nm using a using ε_280_ = 9065 M^−1^cm^−1^ and ε_280_ = 9065 M^−1^cm^−1^ for *Lm*PDT and *Ec*HPRT, respectively.

### 3.3. Enzyme Immobilization

*Lm*PDT and *Ec*HPRT were immobilized separately onto MagReSyn^®^NTA magnetic microbeads (ReSyn Biosciences, Pretoria, South Africa). For this procedure, 20 μL of commercial microparticles suspensions (25 mg/mL) were washed and equilibrated three times with 200 μL binding buffer (80 mM phosphate buffer, 40 mM imidazole, 1 M NaCl, pH 8.0). Subsequently, immobilization was carried out in a final volume of 200 μL using different amounts of *Lm*PDT (60–100 μg) or *Ec*HPRT (60–140 μg). The enzyme solution with the binding buffer was adjusted to 10 times the volume of the initial beads suspension. The enzyme and beads suspensions were incubated for 20 min at room temperature in a roller shaker from J.P.Selecta (Barcelona, Spain). After the binding, the supernatant was removed with the assistance of a magnet and stored for catalyst load determination. Then, microparticles were washed three times with washing buffer (80 mM phosphate buffer, 1 M NaCl, pH 8.0) to remove non-affinity bound enzymes. Finally, beads were resuspended in 200 μL of 10 mM phosphate buffer pH 7.0 (for *Lm*PDT immobilization) and 200 μL of 20 mM Tris-HCL buffer pH 8.0 (for *Ec*HPRT) and stored at 4 °C. Immobilization yields were determined by SDS-PAGE densitometry, based on the difference between protein blanks and immobilization supernatants, using a ChemiDoc unit (Bio-Rad) and Image Lab^TM^ software.

### 3.4. N-Deoxyribosyltransferase Assay

The standard assay for soluble *Lm*PDT was carried out using 0.3 μg of the pure enzyme with 10 mM substrates (dIno and Ade) in 50 mM MES buffer, pH 6.5 (reaction volume 40 μL) at 40 °C for 5 min (300 rpm). Then the reaction was stopped as previously described [33] and the samples were half-diluted with water for HPLC analysis. One unit of activity (U) was established as the amount of enzyme (mg) that produces 1 μmol of dAdo per min (IU).

The standard assay for M*Lm*PDT derivatives was carried out using 0.6 μg of the immobilized enzyme under the aforementioned reaction conditions in a final volume of 80 μL. For immobilized derivatives, one unit of activity (U) was established as the amount of derivative (g) that produces 1 μmol of product per min (IU).

All the determinations were made in triplicate, with the highest error being less than 5%.

### 3.5. Phosphoribosyltransferase Activity Assay

The standard phosphoribosyltransferase activity assay for soluble *Ec*HPRT was performed by incubating 0.25 µg of the pure enzyme with a 40 µL solution containing 10 mM PRPP, 10 mM Hyp, 12 mM MgCl_2_ in 12 mM Tris-HCl pH 8 at 50 °C and 300 r.p.m. for 5–10 min. After this, the enzyme was inactivated as previously described [12], and the IMP production was analyzed and quantitatively measured using HPLC. All determinations were carried out in triplicate, and the maximum error was less than 5%. Under such conditions, one activity unit (U) was defined as the amount of enzyme (mg) producing 1 μmol/min (IU) of IMP under the assay conditions.

Like M*Lm*PDT, the standard assay for M*Ec*HPRT was adapted from the activity assay of the soluble enzyme. Accordingly, 0.5 μg of the immobilized enzyme was incubated under the aforementioned reaction conditions in a final volume of 80 μL. For immobilized derivatives, one unit of activity (U) was established as the amount of derivative (g) that produces 1 μmol of IMP per min (IU).

All the determinations were made in triplicate, with the highest error being less than 5%.

### 3.6. Biochemical Characterization of Immobilized Derivatives

To establish the optimal operational conditions for the M*Lm*PDT and M*Ec*HPRT derivatives, the effect of pH and temperature on the activity of the biocatalyst was assayed. The influence of pH on catalyst activity was tested using different reaction buffers (50 mM sodium citrate, pH 3–6; 50 mM MES, pH 6–7; 50 mM Tris-HCl, pH 7–9; 50 mM sodium phosphate, pH 6–8; 50 mM sodium borate, pH 8–10) under standard assay conditions. Similarly, the effect of temperature on catalyst activity was assayed across the 20–80 °C range.

### 3.7. MLmPDT3/MEcHPRT3 Standard Assay

To deepen the potential applicability of M*Lm*PDT3/M*Ec*HPRT3 as catalysts for the industrial production of nucleosides, the enzyme-mediated synthesis of cladribine was selected as a reaction of interest. In this respect, the reactions were carried out by incubating 0.6 μg of immobilized *Lm*PDT (M*Lm*PDT3) and 0.2 μg of immobilized *Ec*HPRT (M*Ec*HPRT3) with 1 mM dIno, 1 mM 2-Clade, 1 mM PRPP, and 1.2 MgCl_2_, in 50 mM Tris-HCl, pH 7.0, in a final volume of 80 μL, with 300 rpm orbital shaking for 20 min.

All of the determinations were made in triplicate, with the highest error being less than 5%.

### 3.8. Biochemical Characterization of the MLmPDT3/MEcHPRT3 System

The effects of pH and temperature on the activity and stability of the multi-enzymatic system were also assayed to determine the optimal conditions. On one hand, the effect of pH on catalyst activity was assayed using various reaction buffers (50 mM sodium citrate, pH 3–6; 50 mM MES, pH 6–7; 50 mM Tris-HCl, pH 7–9; 50 mM sodium phosphate, pH 6–8; and 50 mM sodium borate, pH 8–10) under standard assay conditions. On the other hand, the temperature dependence of M*Lm*PDT3/M*Ec*HPRT3 was evaluated across the 20–80 °C range. 

### 3.9. Thermal Inactivation of the MLmPDT3/MEcHPRT3 System

Once the optimal conditions were determined, the long-term operational stability of the M*Lm*PDT3/M*Ec*HPRT3 system was also assayed by incubating both derivatives simultaneously at 40 °C and 50 °C for 24 h in 50 mM Tris-HCl, pH 7. Periodically, aliquots were taken, and the activity was measured under the optimal conditions determined after the biochemical characterization.

### 3.10. Application of the MLmPDT3/MEcHPRT3 System in the Manufacturing of Cladribine and IMP

To evaluate the potential applicability of the M*Lm*PDT3/M*Ec*HPRT3 system on cladribine synthesis, different operational aspects were studied. Firstly, a comparative study of enzyme-mediated synthesis of cladribine catalyzed by M*Lm*PDT3 and M*Lm*PDT3/M*Ec*HPRT3 was proposed under similar conditions. To this end, 0.6 μg of immobilized *Lm*PDT (or 0.6 μg immobilized *Lm*PDT and 0.2 μg of immobilized *Ec*HPRT), was incubated with 1 mM dIno, 1 mM 2-ClAde (including 1 mM PRPP and 1.2 MgCl_2_ for the multi-enzymatic system), in 50 mM Tris-HCl, pH 7.0, in a final volume of 80 μL, with 300 rpm orbital shaking for 20 min. After demonstrating the benefits of using the M*Lm*PDT3/M*Ec*HPRT3 system instead of M*Lm*PDT3, diverse time-course reaction curves at different M*Lm*PDT3/M*Ec*HPRT3 ratios were performed for the optimization of the bioprocess.

Finally, the reusability of the multi-enzymatic system was studied by using the magnetic derivatives for 21 consecutive 10 min reactions in cladribine synthesis. After each use, the magnetic derivatives were recovered by an imposed magnetic field and washed with 50 mM Tris-HCl pH 7.0. Then, the recovered derivatives were introduced into a fresh reaction medium and reused again.

### 3.11. Analytical Methods

The quantitative determination of products was performed by HPLC analysis using an ACE EXCEL 5 μm CN-ES 250 × 4.6 mm equilibrated, with 0.1 M triethyl ammonium acetate at a flow rate of 0.8 mL/min. Retention times for natural bases, nucleosides, and NMPs (abbreviated according to the recommendations of the IUPAC) were as follows; Hypoxanthine (Hyp), 9 min; 2′-deoxyinosine (dIno), 10.8 min; inosine-5′-monophosphate (IMP), 5.3 min; 2-chloroadenine (2-ClAde), 15.8 min; cladribine, 18.5 min; adenine (Ade): 10 min; and 2′-deoxyadenosine (dAdo), 15 min.

## 4. Conclusions

Herein, the functional characterization of an immobilized cascade system for cladribine manufacturing based on the sequential actions of two magnetic biocatalysts is presented. Moreover, the application of this multi-enzymatic system also demonstrated other advantages, such as the minimization of undesired by-products, the generation of other added-value molecules, the shifting of the equilibrium of the transglycosylation, and easy separation of the catalysts from the reaction medium.

## Figures and Tables

**Table 1:** Enzymatic synthesis of cladribine M*Lm*PDT3 or M*Lm*PDT3/M*Ec*HPRT.

	Reaction 1				Reaction 2				
	Substrates		Product	Conversion (%)		Substrates		Product	Conversion (%)
	dIno [mM]	2-ClAde [mM]	Cladribine [mM]			Hyp [mM]	PRPP [mM]	IMP [mM]	
M*Lm*PDT3 ^a^	1	1	0.40 ± 0.03	40		-	-	-	-
M*Lm*PDT3/M*Ec*HPRT3 ^b^	1	1	0.52 ± 0.04	52		0.52	1	0.39 ± 0.01	75

^a^ Reaction conditions: 0.6 μg of immobilized *Lm*PDT in 80 μL at 40 °C, 20 min and 300 r.p.m. [dIno] = [2-ClAde] = 1 mM, in 50 mM Tris-HCl, pH 7.0. ^b^ Reaction conditions: 0.6 μg of immobilized *Lm*PDT, 0.2 μg of immobilized *Ec*HPRT in 80 μL at 40 °C, 20 min and 300 r.p.m. [dIno] = [2-ClAde] = [PRPP] =1 mM, [MgCl_2_] = 1.2 mM in 50 mM Tris-HCl, pH 7.0.

## Supplementary Materials

The following supporting information can be downloaded at: https://www.mdpi.com/article/10.3390/ijms232113634/s1.
