# Supplementary material for: Magnetic Multi-Enzymatic System for Cladribine Manufacturing
Source: Int J Mol Sci. 2022 Nov 7;23(21):13634. doi: 10.3390/ijms232113634 (PMC9658597; doi:10.3390/ijms232113634)
Supplement: Supplementary file 1 [file ijms-23-13634-s001.zip › ijms-2005031-supplementary.pdf]

International Journal of *Molecular Sciences*

Magnetic Multi-Enzymatic System for Cladribine Manufacturing

Guillermo Cruz [1], Laura Pilar Saiz [1], Muhammad Bilal [2], Lobna Eltoukhy [3], Christoph Loderer [3,*] and Jesús Fernández-Lucas [1,*]

[1] Applied Biotechnology Group, Universidad Europea de Madrid, Urbanización El Bosque, Calle Tajo, s/n, 28670 Villaviciosa de Odón, Spain
[2] Institute of Chemical Technology and Engineering, Faculty of Chemical Technology, Poznan University of Technology, Berdychowo 4, PL-60695 Poznan, Poland
[3] Chair of Molecular Biotechnology, Institute for Microbiology, Technische Universität Dresden, Zellescher Weg 20b, 01217 Dresden, Germany
* Correspondence: christoph.loderer@tu-dresden.de (C.L.); jesus.fernandez2@universidadeuropea.es (J.F.-L.)

Electronic Supplementary Information

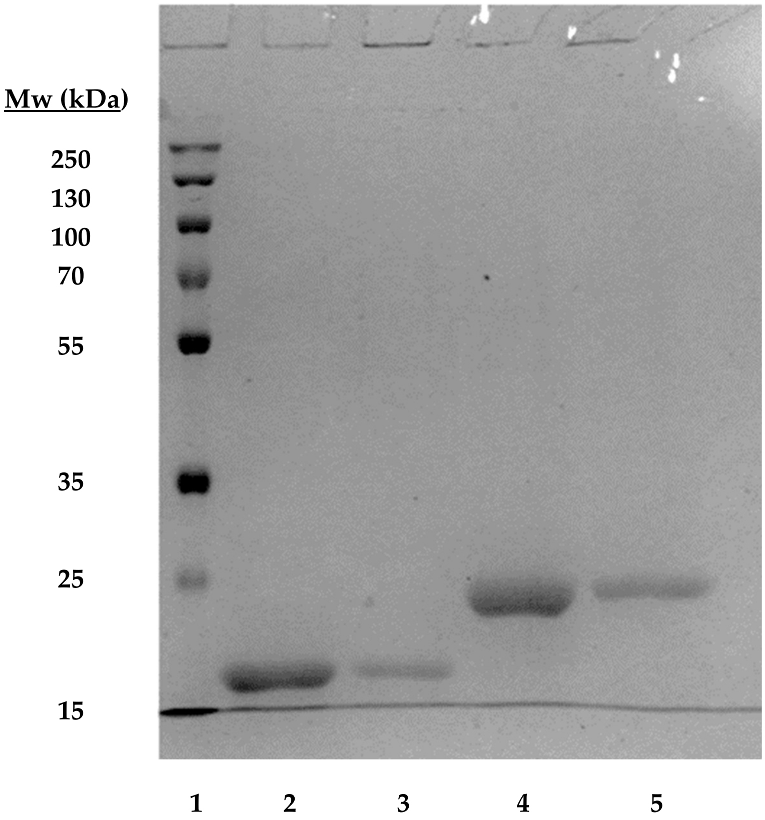

Figure S1. SDS-PAGE analysis of pure *Ld*NDT. **Lane 1.** Prestained standard proteins (Thermoscientific used as molecular weight markers. **Lane 2-3.** Pure fractions of soluble *Ld*NDTwt. **Lane 4-5.** Pure fractions of soluble *Ec*HPRT. *Ld*NDT: NDT from *Lactobacillus delbrueckii*; *Ec*HPRT: HPRT from *E. coli.*

Tables

Table S1. Effect of enzyme/support mass ratio on the immobilization of *Lm*PDT and *Ec*HPRT onto MagReSyn®NTA microspheres.

Derivative	Added $mg_{enz}/g_{support}$	Immobilization Yield (%)	Biocatalyst Load ($mg_{enz}/g_{support}$)	Activity ($IU/g_{support}$)	Recovery (%)
***Lm*PDT**[a]					
M*Lm*PDT1	120	100	120	8425 ± 384	55
M*Lm*PDT2	160	98	157	11023 ± 502	57
M*Lm*PDT3	200	85	170	11935 ± 544	63
***Ec*HPRT**[b]					
M*Ec*HPRT1	120	100	120	8414 ± 123	45
M*Ec*HPRT2	160	100	160	10989 ± 311	44.5
M*Ec*HPRT3	200	90	180	12840 ± 484	45

[a] Reaction conditions: 0.6 μg of immobilized *Lm*PDT, [dIno] = [Ade] = 10 mM, in 50 mM MES buffer, pH 6.5 at 40 °C, 5 min, 300 r.p.m. Vr = 80 μL. [b] Reaction conditions: 0.5 μg of immobilized *Ec*HPRT, [Hyp] = [PRPP] = 10 mM, [$MgCl_2$]=12 mM, in 12 mM Tris-HCl buffer, pH 8 at 50 °C, 5-10 min, 300 r.p.m. Vr = 80 μL.
